# Polyphenols Sensitization Potentiates Susceptibility of MCF-7 and MDA MB-231 Cells to Centchroman

**DOI:** 10.1371/journal.pone.0037736

**Published:** 2012-06-29

**Authors:** Neetu Singh, Deeba Zaidi, Hari Shyam, Ramesh Sharma, Anil Kumar Balapure

**Affiliations:** Tissue and Cell Culture Unit (TCCU), CSIR-Central Drug Research Institute, Council of Scientific & Industrial Research, Lucknow, India; Sudbury Regional Hospital, Canada

## Abstract

Polyphenols as “sensitizers” together with cytotoxic drugs as “inducers” cooperate to trigger apoptosis in various cancer cells. Hence, their combination having similar mode of mechanism may be a novel approach to enhance the efficacy of inducers. Additionally, this will also enable to achieve the physiological concentrations facilitating significant increase in the activity at concentrations which the compound can individually provide. Here we propose that polyphenols (Resveratrol (RES) and Curcumin (CUR)) pre-treatment may sensitize MCF-7/MDA MB-231 (Human Breast Cancer Cells, HBCCs) to Centchroman (CC, antineoplastic agent). 6 h pre-treated cells with 10 µM RES/CUR and 100 µM RES/30 µM CUR doses, followed by 10 µM CC for 18 h were investigated for Ser-167 ER-phosphorylation, cell cycle arrest, redox homeostasis, stress activated protein kinase (SAPKs: JNK and p38 MAPK) pathways and downstream apoptosis effectors. Low dose RES/CUR enhances the CC action through ROS mediated JNK/p38 as well as mitochondrial pathway in MCF-7 cells. However, RES/CUR sensitization enhanced apoptosis in p53 mutant MDA MB-231 cells without/with involvement of ROS mediated JNK/p38 adjunct to Caspase-9. Contrarily, through high dose sensitization in CC treated cells, the parameters remained unaltered as in polyphenols alone. We conclude that differential sensitization of HBCCs with low dose polyphenol augments apoptotic efficacy of CC. This may offer a novel approach to achieve enhanced action of CC with concomitant reduction of side effects enabling improved management of hormone-dependent breast cancer.

## Introduction

Anti-estrogen therapies constitute the mainstay of current treatment for breast cancer [1]. Centchroman (CC), a triphenylethylene Selective Estrogen Receptor Modulator (SERM) has been considered a potential anti-breast cancer drug in ER-positive (MCF-7)/−negative (MDA MB-231) Human Breast Cancer Cells (HBCCs) as well as with all stages of hormone-responsive breast cancer [2]–[4]. Studies have demonstrated that low concentrations of triphenylethylenes in patients cause modest, partial estrogen-like action [5]. Although once a steady level is achieved the drug shows its anti-estrogenic properties whose attainment requires 4–8 weeks [5]. Therefore, to overcome the low dose estrogenic effects of triphenylethylenes, one alternative approach would be to sensitize the cells through supplementing compounds (polyphenols) having similar mode of action. This in turn enables achievement of physiological levels facilitating significant increase in the activity at concentrations which the compound can individually provide. For this reason, polyphenols like Resveratrol (RES) abundant in wine and *Curcumin longa* derived Curcumin (CUR) a South Asian and Middle Eastern spice were selected as Type I estrogens similar to CC for preconditioning the cells [5]. Moreover, 2–3 times higher retention of polyphenols in local tissue as compared to the plasma levels [nM to low µM (∼2 µM ); retention time ∼5–6 h] [6], [7] may positively influence the cell susceptibility to triphenylethylenes.

Therefore, we pre-treated estrogen-deprived CC treated MCF-7/MDA MB-231 cells with physiological and pharmacological doses of polyphenols to synergize or antagonize the drug action. Both, polyphenols (CUR and RES) and CC bind to ERα and β with lower affinity than E2 [8], [9] involving ER-dependent and -independent pathways [2], [10]–[13]. Hence, sensitization with low dose polyphenols may modulate the receptor levels/redox status overtly improving CC efficacy. Contrarily, high dose polyphenols may disrupt ER and enhance the ER-independent antiproliferative effect in the combination. This may modulate proapoptotic (Bax, Caspase-9), versus antiapoptotic genes (Bcl-2) ratio and transcription factors p53, including c-Jun and phospho-ATF-2 to play a crucial role in sensitizing the caspase-3 deleted and p53 mutant MCF-7 and MDA MB-231 respectively.

Hence, we investigated the mode of action of polyphenol-sensitized, CC treated MCF-7/MDA MB-231 cells on cell cycle, redox homeostasis, stress activated protein kinase (SAPKs: JNK and p38 MAPK) and downstream apoptosis effectors. We also employed a series of inhibitors for p53, JNK and p38 pathways critical for time-dependent cytotoxicity of CC and polyphenols. Both RES and CUR at physiological dose potentially sensitize CC-induced apoptosis in MCF-7 cells through modulating the above factors. Pharmacological sensitization significantly arrests the cells (Go/G1 phase; RES and G2/M phase; CUR) rescuing them from CC-induced apoptosis through unaltered factors to the same extent as polyphenols alone. However, on sensitization with low dose RES (JNK/p38-independent)/CUR (JNK/p38-dependent), the apoptosis enhanced in CC treated MDA MB-231 cells. Contrarily, with high dose polyphenol sensitization CC in both the combinations showed JNK/p38-dependent apoptosis. This therefore reinforces our hypothesis about the usage of polyphenols to ameliorate Estrogen-dependent/independent breast cancer with enormous translational potential during combination chemotherapy employing Centchroman.

## Materials and Methods

### Materials

Dulbecco’s Modified Eagle’s Medium (DMEM) with or without phenol red, N-[2-Hydroxyethyl] piperazine-N′-2-ethanesulfonic acid (HEPES), Penicillin G, Streptomycin sulfate, Gentamicin sulfate, Dulbecco’s Phosphate Buffered Saline (PBS), Propidium Iodide (PI), Ribonuclease-A, inhibitors specific to JNK (SP600125), p38 (SB203580) and pifithrin (Pif) were purchased from Sigma Chemical Co., St. Louis, MO, USA. 2′, 7′-Dichlorofluorescin Diacetate (DCFDA) from Merck–Calbiochem whereas Fetal Calf Serum (FCS) was procured from GIBCO BRL Laboratories, New York, USA. 5,5′,6,6′-tetrachloro-1,1′,3,3′-tetraethyl-benzimidazolylcarbocyanine Chloride (JC-1) was purchased from BioVision Research Products (Mountain View, CA, SA). Protease and Phosphatase inhibitor cocktails, Cell Lysis M ™ Cell Lysis reagent, antibodies against β-actin, p53, phospho (Ser-15) p53, MnSOD, Catalase, non-activated p38, phosphor Ser-167 AF-1 region of ERα and ExtrAvidin® Alkaline Phosphatase Staining kit as well as BCIP/NBT tablets were from Sigma Chemical Co. (St. Louis, MO, USA). Antibodies against Bcl-2, Bax, JNK 1/2 were purchased from BioVision Research Products (Mountain View, CA, SA). p38 MAPK Kinase Assay Kit was from Cell Signalling whereas Kinase Star ™ JNK Activity Assay Kit was procured from Biovision. All other chemicals used were of analytical grade.

### Cell Culture

MCF-7/MDA MB-231 (Human Breast Cancer, epithelial) cells were procured from the National Center for Cell Sciences (NCCS), Pune, India. These cells were routinely cultured as reported previously by us [14], [15]. Briefly, the cells were cultured in DMEM pH 7.4, containing Penicillin (200 µg/mL), Streptomycin (100 µg/mL), Gentamicin (60 µg/mL) supplemented with 10% FCS and 10 mM HEPES in a humidified 5% CO_2_ incubator at 37°C.

### Treatments

For experimental purpose, the cells from a 70–80% confluent flask were trypsinized and cultured for a total of 3 days. Initially for the first 2 days, the cells were conditioned in phenol red-free DMEM containing Penicillin (200 µg/mL ), Streptomycin (100 µg/mL), Gentamicin (60 µg/mL), L-Glutamine (2 mM), D-Glucose(1 g/L), Sodium pyruvate (0.11 g/L), HEPES (10 mM) supplemented with 10% Dextran Coated Charcoal stripped FCS (DCC/FCS) (2). Subsequently, the cells were pre-treated with polyphenols (RES: 10/100 µM and CUR: 10/30 µM) for 6 h and continued with CC (10 µM) for next 18 h (total of 24 h). Polyphenols, SP600125 and SB203580 were dissolved in dimethyl sulphoxide (DMSO). Controls were treated with 0.1% DMSO. The cell number used for each experiment has been described individually.

### Inhibitor Treatments

ROS inhibition was performed using N-Acetyl-l-Cysteine (L-NAC, 5 mM) 1 h prior to treatment with drugs. For JNK/p38 pathway inhibition, during the 6 h exposure to polyphenols (RES: 10/100 µM and CUR: 10/30 µM), JNK inhibitor (10 µM SP600125)/p38 pathway inhibitor (1 µM SB203580)/p53 pathway inhibitor (15 µM Pif) was added during the last two hours prior to CC exposure. The last regimen with CC was continued until 18 h. The cells were analyzed by FACS (PI 40 µg/mL), western blotting and immunoprecipitation. These concentrations of inhibitors (L-NAC, SP6000125, SB203580 and Pif) were nontoxic to cells under our pre-incubation conditions (data not shown).

### Cellular Morphological and Cell-Cycle Analysis

For time-dependent morphological analysis, 0.2×10^6^cells were seeded in a 6-welled plate and treated for 3, 6 or 24 h with polyphenols (RES: 10/100 µM and CUR: 10/30 µM) or preconditioned with polyphenols for 6 h and continued with CC (10 µM) for next 18 h. These cells were then photographed by a Nikon Eclipse Ti inverted phase contrast microscope. Cells were also stained with 40 µg/mL PI prior to analysis by a FACS calibur instrument (Becton Dickinson, San Jose, CA, USA) employing the Cell Quest Software [15].

### Modulation of Mitochondrial Membrane Potential (Δψm) and Intracellular Reactive Oxygen Species (ROS) Generation

Mitochondrial Membrane Potential (Δψm) was measured by the uptake of JC-1 which is a dye that stains mitochondria in living cells in a membrane potential dependent fashion. In apoptotic cell, the dye stays in the cytoplasm as a monomer and fluoresces green (527 nm), while in healthy cells, the dye aggregates in the mitochondria and fluoresces red (590 nm). For the experiment, 0.2×10^6^ cells were plated in a 6-welled plate, treated as above, washed and finally harvested in chilled PBS containing 1 µM JC-1. The samples were incubated at 37°C for 30 min in dark, washed twice with PBS and fluorescence intensities were determined on a Flow Cytometer [15].

Cells were seeded at a concentration of 10×10^3^ cells per well in black 96-well plates. Cells were treated time-dependently with polyphenols or pre-conditioned with polyphenols for 6 h and continued with CC (10 µM) for next 18 h (total of 24 h). This was followed by incubation with 10 µM 2′,7′-dichlorofluorescin diacetate (DCFDA) (Molecular Probes, Eugene, OR) for 30 min. Cells were then rinsed with PBS and then placed in a pre-warmed 37°C fluorimeter for time-dependent measurement (DCFDA Ex 480 nm, Em 530 nm). Following this, the supernatant was removed and a protein assay was performed on the cells. The final results have been corrected for variations in the protein concentration between wells and are expressed as a percentage activity in untreated controls being 100%. Flow cytometry analysis of cells stained with DCFDA was performed to confirm results using 5 mM L-NAC. HBCCs were plated in 6-well plates and treated time-dependently with polyphenols or pre-treated with polyphenols accompanied in the presence of DCFDA (10 µM). Fluorescence was measured with a Flow Cytometer (Ex 500 nm, Em 530 nm) [15].

### Determination of Total Glutathione (GSH)

Quantification of total GSH was accomplished using a Glutathione Assay Kit from Sigma. According to the manufacturer’s instructions, 1×10^8^ cells were exposed as above and deproteinized with 5% 5-Sulfosalicylic Acid solution after three rounds of alternate freeze-thawing, centrifuged to remove the precipitated protein and the supernatants assayed for GSH. The kit uses a kinetic assay in which catalytic amounts (nM) of GSH cause a continuous reduction of 0.031 mg/mL DTNB (5,5′-dithiobis (2-nitrobenzoic acid) to TNB (monitored at 412 nm in a SpectraMax M2 Multiwell Plate Reader). The GSSG formed is reduced by glutathione reductase (0.115 units/mL) and 48 µM NADPH to additionally give a positive value. The amount of GSH was calculated from a standard curve generated under similar conditions.

### Superoxide Dismutase and Catalase Activities

Superoxide dismutase (SOD) and Catalase (CAT) activities were determined according to McCord and Fridovich, and Beutler [16], [17] respectively. Untreated as well as treated 3×10 ^6^ MCF-7/MDA MB-231 cells were collected by scraping, centrifuging and the pellets sonicated in chilled 1.15% KCl. The cell extract was spun at 1500×g for 5 min at 4°C. The resulting supernatants were analyzed for enzyme activities. The specific activity of SOD was calculated as follows: Specific Activity  =  A×dilution factor×1000/One unit×volume of enzyme (µl)×mg protein. “A” = OD change/min of controlled reaction-OD change/min of experiment, “One unit” = (OD change/min of controlled reaction)/2. Here, one unit means 50% reduction in NBT reduction as compared to control. The specific activity of CAT was calculated by the following equation: Specific Activity  =  OD change/min×dilution factor×1000/0.00394×volume of enzyme (µl)×mg protein  =  µmol H_2_O_2_ reduced/min/mg protein. Each data point was performed in triplicate and the results reported as Mean Absorption ± SD.

### Western Blotting

3×10^6^ cells were cultured in T-75 flasks and treated as above. Pellet was disrupted in lysis buffer procured from Cell Lysis M ™ Cell Lysis reagent, Sigma Chemical Co. (St. Louis, MO, USA) supplemented with protease and phosphatase inhibitor cocktail as per manufacturer’s protocol. The supernatant was assayed for protein content [18]. 50 µg of protein per lane were processed for western blotting by SDS-PAGE and electro-transferred to nitrocellulose membranes. Membranes were probed with the appropriate primary antibodies against MnSOD, Catalase, Total p53, phospho p53 (Ser-15), JNK 1/2, non-activated p38, Bcl-2, Bax, active Caspase-9 and Ser-167 phosphorylation of AF-1 region of ERα. All primary antibodies were used at recommended dilution in 1% bovine serum albumin in Tris buffered saline with 0.1% Tween™ 20. Reactions were visualized with biotin conjugated secondary antibody and subsequently with avidin linked Alkaline-Phosphatase enzyme conjugate according to the manufacturer’s protocol. Immuno-detection was accomplished using insoluble colored substrate BCIP/NBT.

### Assay for JNK and p38 Activity

KinaseStar ™ JNK Activity Assay Kit (BioVision) was used to assess JNK activity according to the manufacturer’s instructions. Briefly, activated JNK was immunoprecipitated by incubating cell lysates for 45 min with an immobilized JNK specific antibody at room temp. The *in vitro* kinase assay was then performed using c-Jun protein/ATP mixture as substrate. Levels of phosphorylated c-Jun were then detected by western blotting using an anti phospho-c-Jun (Ser 73) antibody.

p38 activity was assessed using a non-radioactive p38 MAP Kinase Assay Kit (Cell Signalling) according to the manufacturer’s instructions. Briefly, phosphorylated p38 was immunoprecipitated by incubating cell lysates overnight with an immobilized phospho-p38 antibody at 4°C. An *in vitro* kinase assay was then performed using an ATF-2 fusion protein as substrate. Levels of phosphorylated ATF-2 were then detected by western blotting using a phospho-ATF-2 (Thr71) antibody.

### Statistical Analysis

The results are expressed as Mean ± SD from one of three similar experiments each performed in triplicate. Student’s ‘t-test’ was used to determine the level of significance and the following values assigned * P<0.05; ψ P<0.01; # P<0.001, calculated compared to control unless stated otherwise.

## Results

### Morphological and Cell-Cycle Changes in Polyphenols *per se* and Polyphenols Sensitized CC Treated MCF-7/MDA MB-231 Cells

**Figure 1:**
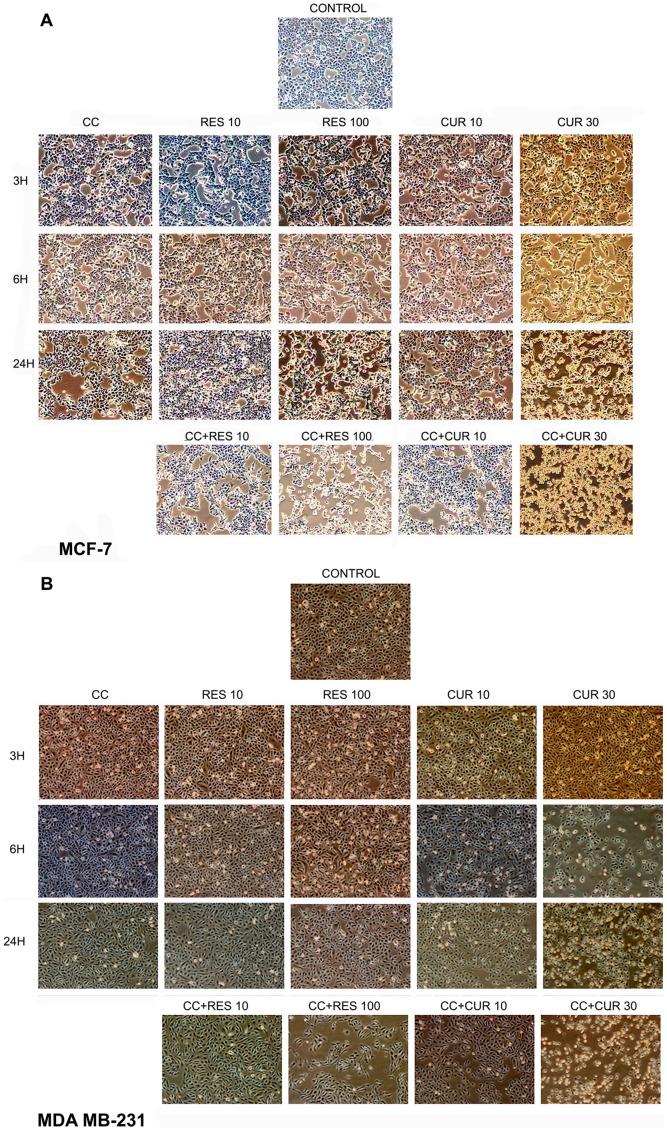
Time-dependent morphological analysis. 0.2×10^6^cells were seeded in a 6-welled plate and treated for 3, 6 and 24 h with polyphenols (RES: 10/100 µM and CUR: 10/30 µM) and CC (10 µM) alone or preconditioned with low/high dose polyphenols for 6 h and continued with CC (10 µM) for next 18 h {A (MCF-7) and B (MDA MB-231)}. The cells were then photographed by a Nikon Eclipse Ti inverted phase contrast microscope. Data shown is one of the three similar experiments each performed in triplicate.

Time-dependent, cytomorphological analysis shows typical epithelial morphology at low doses of polyphenol treated MCF-7/MDA MB-231 cells as compared to control at all time points. Adversity begins in high doses of polyphenols as early as 6 h and reaches to its maximum at 24 h in MCF-7 (Fig. 1A). While in MDA MB-231 cells, 30 µM CUR displays changes as early as 6 h until 24 h. However, 100 µM RES displays changes only at 24 h (Fig. 1B). CC alone time-dependently deteriorates the morphology, evident by decrease in cell density in MCF-7 cells which was restricted to 24 h time point in the other cell type (Fig. 1A and B).

At 24 h, low dose polyphenol sensitizes CC treated cells showing higher degree of distortion compared to either drug alone. On sensitizing with high doses of polyphenols, CC depicts adverse morphology as compared to CC and polyphenols alone in both the cells (Fig. 1A and B).

**Figure 2:**
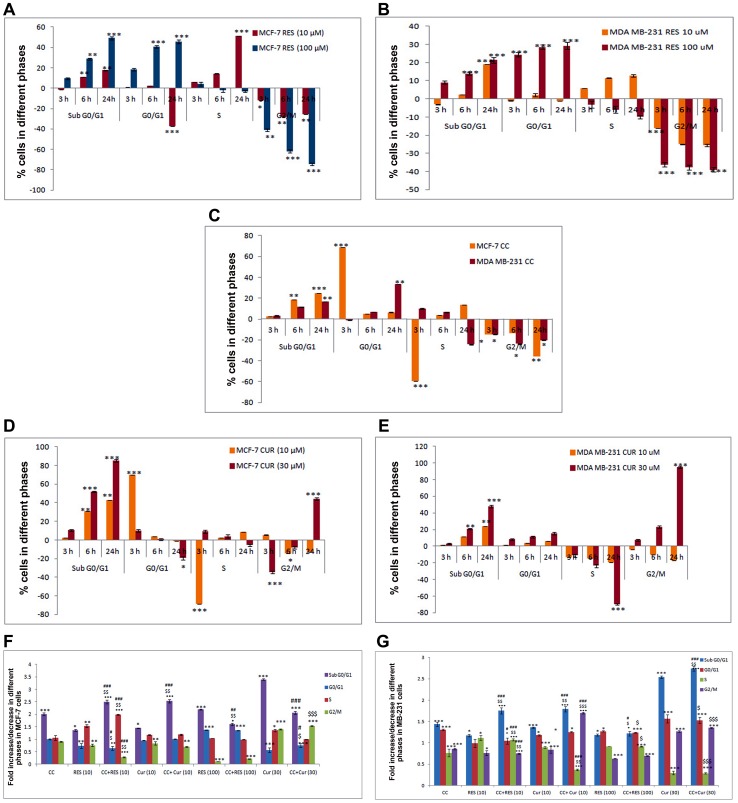
Cell-Cycle analysis of RES, CUR and CC treated MCF-7 cells (Time-dependent and sensitized). Briefly, 0.2×10^6^ cells were pre-cultured for 48 h in phenol red-free DMEM then exposed for 3, 6 and 24 h with 10 µM/100 µM RES {A (MCF-7), B (MDA MB-231)}, 10 µM CC (C), 10 µM/30 µM CUR {D (MCF-7), E (MDA MB-231)} or preconditioned cells with polyphenols (RES: 10/100 µM and CUR: 10/30 µM) for 6 h and continued with CC (10 µM) for next 18 h (total of 24 h). Following incubations the MCF-7 (F) and MDA MB-231 (G) cells were harvested, permeabilized, stained with Propidium Iodide (40 µg/mL) and analyzed by Flow Cytometer (employing Modfit Software); % CV <10. Data shown are the mean ± S.E. of one of the three similar experiments each performed in triplicate. * P<0.05; ** P<0.01; *** P<0.001, calculated compared to control. $ P<0.05; $$ P<0.01; $$$ P<0.001 with respect to individual CC dose and # P<0.05; ## P<0.01; ### P<0.001 with respect to individual RES dose.

To determine whether polyphenols pre-treatment augments apoptosis in CC treated MCF-7/MDA MB-231 cells, a sub-G0/G1 DNA peak, suggestive of apoptotic DNA was tested. However, the number of cells in this population on treatment with 10 µM RES increased significantly with 6 h which extended up to 24 h. Even though the cells were undergoing apoptosis, they passed from G1 to S and G2 phase. The transition of cells from S to G2/M phase was blocked within 6 h and the inhibition persisted up to 24 h, the time point at which most of the cells were arrested in S phase. With higher dose of RES, the cells were arrested in G0/G1 phase as early as 3 h and continued up till 24 h. This goes on along with unaltered S phase and time-dependent decrease in G2/M phase (Fig. 2A). In MDA MB -231 cells, 10 µM RES showed increased cell death by 24 h accompanied with decreased G2/M phase. However, it failed to evoke any S-phase arrest compared to MCF-7 cells. Conversely, 100 µM RES showed equivalent sub-G0/G1 compared to 10 µM RES, significant accumulation of G0/G1 and decline in G2/M cells, initiated at 3 h and continued up till 24 h (Fig. 2B).

CC when added to the culture separately, the transition of cells from G1 to S phase was blocked within 3 h but with longer treatment, the inhibition was released and the cells started cycling again accompanied with apoptosis in MCF-7 cells. Time-dependent increase in sub-G0/G1 cells was observed in MDA MB-231 cells (Fig. 2C). The cell number in sub-G0/G1 population increased with time up to 24 h with 10 µM CUR treatment with concomitant decrease in G0/G1 population in MCF-7 cells (Fig. 2D), while remaining unaltered G0/G1 in MDA MB-231 cells (Fig. 2E). With 30 µM CUR, the cells undergoing apoptosis passed from G1 to S and G2/M phases with final arrest in G2/M phase at 24 h in both the MCF-7/MDA MB-231 cells (Fig. 2D and E).

MCF-7 cells pre-treatment with 10 µM RES for 6 h, CC significantly up-regulates sub-G0/G1 peak, S phase arrest and down-regulates G0/G1, G2/M phase than either drug alone (Fig. 2F). For MDA MB-231 cells under similar conditions, higher sub-G0/G1 and decreased G2/M was observed (Fig. 2G). In 100 µM RES sensitized CC treated cells G0/G1, S and G2/M phases mirrored with 100 µM RES apart from significant decline in sub-G0/G1 population in MCF-7 cells and unaltered in MDA MB-231 cells (Fig. 2F and G). Upon comparing with CC alone, the G0/G1 arrest improved whereas sub-G0/G1, G2/M phases showed remarkable decline in MCF-7 cells and remain unchanged for MDA MB-231 cells (Fig. 2F and G). With 10 µM CUR sensitization, CC significantly augments sub-G0/G1 peak leaving the rest unaltered as compared to individual drugs *per se* in MCF-7 (Fig. 2F). Interestingly, 10 µM CUR sensitized CC treated MDA MB-231 cells displayed G2/M phase arrest with enhanced sub-G0/G1 peak as compared to drugs alone (Fig. 2G). In 30 µM CUR sensitized CC treated MCF-7 cells, major decline in sub-G0/G1 phase vis-à-vis CUR alone was observed whereas reverse was true for MDA MB-231 cells. On the other hand, G0/G1 and G2/M under similar situations remain unaltered. On comparison with CC, G0/G1 population decreases whereas G2/M phase shows remarkable up-regulation in MCF-7 cells. Significant increase in sub-G0/G1 peak was observed in MDA MB-231 cells (Fig. 2F and G).

### Disruption of Mitochondrial Membrane Potential (ΔΨm) and Reactive Oxygen Species Generation in Polyphenol *per se* and Polyphenol Sensitized CC Treated MCF-7/MDA MB-231 Cells

**Figure 3:**
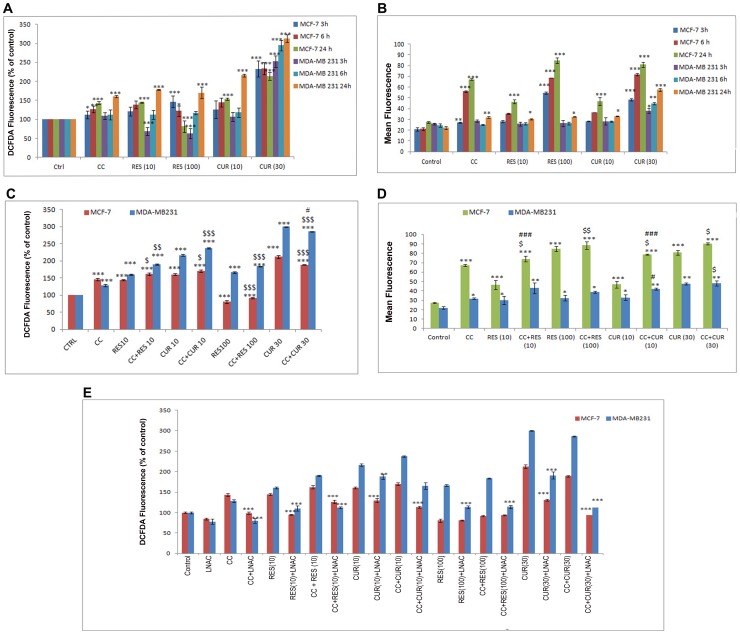
Time-dependent ROS-generation and Mitochondrial Membrane Potential (ΔΨm). 0.2×10^6^ MCF-7/MDA MB-231 cells were plated in 6-well plates, treated time-dependently (3, 6 and 24 h) with CC (10 µM) and polyphenols *per se* {(RES: 10/100 µM and CUR: 10/30 µM; A (ROS-generation) and B (ΔΨm)}; preconditioned cells with low and high dose polyphenols for 6 h and continued with CC (10 µM) for next 18 h {C (ROS-generation) and D (ΔΨm)}. Cells were then rinsed with PBS and then placed in a preheated 37°C fluorimeter for time-dependent measurement of generation of H_2_O_2_ (DCFDA). ΔΨm was determined on a Beckton–Dickinson Fluorescence Activated Cell Sorter. Flow Cytometry analysis of MCF-7 and MDA MB-231 cells stained with DCFDA was performed to confirm ROS results using N-Acetyl-l-Cysteine (L-NAC, 5 mM) 1 hr prior to treatment (E). Data shown are the Mean ± S.E. of one of the three similar experiments each performed in triplicate. * P<0.05; ** P<0.01; *** P<0.001, calculated compared to control. $ P<0.05; $$ P<0.01; $$$ P<0.001 with respect to individual CC dose and # P<0.05; ## P<0.01; ### P<0.001 with respect to individual RES dose.

ROS generation studies were performed through DCFDA-ﬂuorimetric analyses. Low dose polyphenols cause significant increase in ROS at 24 h. This is in agreement with corresponding changes in ΔΨm only at 24 h in MCF-7 cells. Progressive increase in dose-dependent RES-induced ROS generation from 3–24 h was noticed for MDA MB-231 cells with significance only at 24 h accompanied with concomitant disruption of ΔΨm at 24 h. However, dose-dependent CUR in MDA MB-231 cells acted differently inducing ROS and disruption of ΔΨm from 6 through 24 h (Fig. 3A and B). Unlike the low dose polyphenols in MCF-7 cells, 100 µM RES induces ROS at 3 h, with significant reduction at 24 h. Vis-à-vis, 30 µM CUR shows drastic generation of ROS as early as 3 h sustained until 24 h (Fig. 3A). The polyphenols associated dichotomy in cell-cycle owes itself to ROS generation significantly pronounced at the higher dose is a unique observation. As ROS generation is cell-cycle dependent phenomenon [19], [20], RES (100 µM) arrests the cells in G0/G1 phase (3 h) resultantly letting the cells evade the remainder events of the cycle accompanied with minimal ROS generation. Conversely, 30 µM CUR at 3–24 h permits the transition of cells from G1 to S phase to final arrest in G2/M phase owing to persistent ROS generation. Time-dependent gradual decrease in 100 µM RES and persistent ROS generation leads to disruption of ΔΨm in MCF-7 cells [21] (Fig. 3A and B).

However, low dose polyphenols sensitized CC treated cells display increase in ROS and concomitant disruption of ΔΨm than CC alone both in MCF-7 and MDA MB-231 cells (Fig. 3C and D). Conversely, decreased ROS was observed with 100 µM RES sensitized CC treated cells vis-à-vis the ligands *per se* in MCF-7 cells remaining unaltered in MDA MB-231 (Fig. 3C). On addition of CC to 30 µM CUR sensitized cells, ROS generation declined versus CUR but significantly enhanced vis-à-vis CC (Fig. 3C) in both the cell types. The high dose polyphenol combination severely disrupts ΔΨm compared to CC alone in MCF-7 cells only. As compared to high dose polyphenols alone, ΔΨm remained unaltered in both the cell types (Fig. 3D). Further, cytometry reveals that 5 mM L-NAC, inhibits ROS generation by low dose of polyphenols separately or with sensitized cells in MCF-7 and MDA MB-231 cells (Fig. 3E). In MCF-7 cells, L-L-NAC failed to respond with 100 µM RES either or in CC treated whereas it inhibited 30 µM CUR without or with CC generated ROS. Additionally, 100 µM RES/30 µM CUR alone or in CC treated MDA MB-231 cells induced ROS, was also inhabitable by L-L-NAC (Fig. 3E).

### Differential Anti-oxidant Apparatus Expression in Polyphenol Sensitized, CC Treated MCF-7/MDA MB-231 Cells

**Figure 4:**
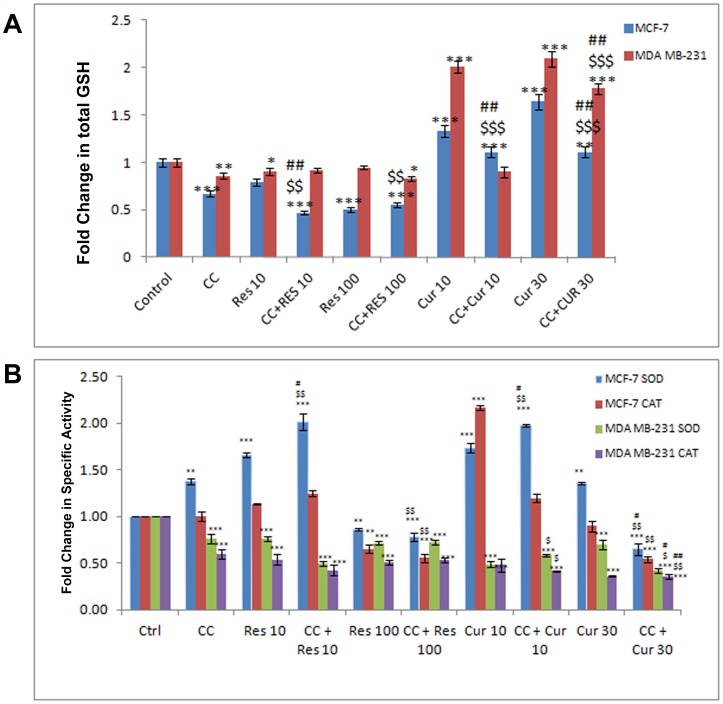
Assessment of antioxidant enzymes (AOs) activity. Quantification of total GSH was accomplished using a Glutathione Assay Kit from Sigma. 1×10^8^ MCF-7/MDA MB-231 cells were exposed to CC (10 µM) and polyphenols *per se* (RES: 10/100 µM and CUR: 10/30 µM) or sensitized with low/high dose polyphenols for 6 h and continued with CC (10 µM) for next 18 h (A). Superoxide dismutase (SOD) and Catalase (CAT) activities were determined according to McCord and Fridovich, and Beutler respectively. Briefly, supernatants obtained from cell extracts of untreated as well as treated 3×10^6^ MCF-7/MDA MB-231 cells were analyzed for enzyme activities (B). Each data point was performed in triplicate and the results reported as Mean Absorption ± SE.

**Figure 5:**
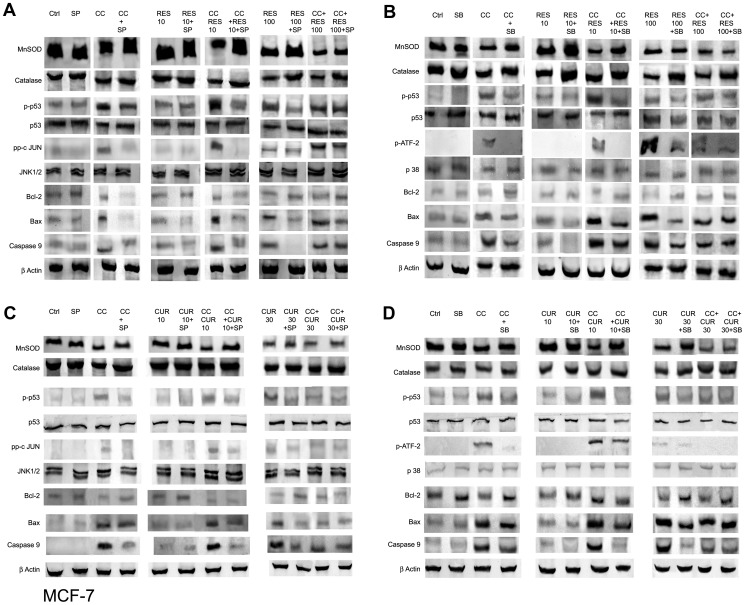
JNK/p38-dependent modulation of expression of Antioxidant enzymes, p53 and apoptogenic factors in MCF-7 cells̀1. For expression analysis, 3×10^6^ MCF-7 cells were pre-cultured for 48 h in phenol red-free DMEM and exposed to CC (10 µM) and polyphenols *per se* {RES: 10/100 µM (A & B) and CUR: 10/30 µM (C & D)} or sensitized with low/high dose polyphenols for 6 h and continued with CC (10 µM) for next 18 h. Using 10 µM of SP600125 (JNK inhibitor)/1 µM of SB 203580 (p38 inhibitor) 2 hours prior to CC exposure, 50 µg of the whole cell lysate was separated on a 10% SDS-PAGE gel, probed with respective antisera following transfer to a nitrocellulose membrane and subsequent immunoblotting. β-actin was used as a protein loading control. (A) RES/CC+RES without and with SP600125; (B) RES/CC+RES without and with SB203580; (C) CUR/CC+CUR without and with SP600125; (D) CUR/CC+CUR without and with SB203580. However, for c-Jun and phospho ATF-2 detection, immunoprecipitation and detection was carried out as detailed in Materials and Methods. Data shown is representative of one of the three similar experiments each performed in triplicate.

**Figure 6:**
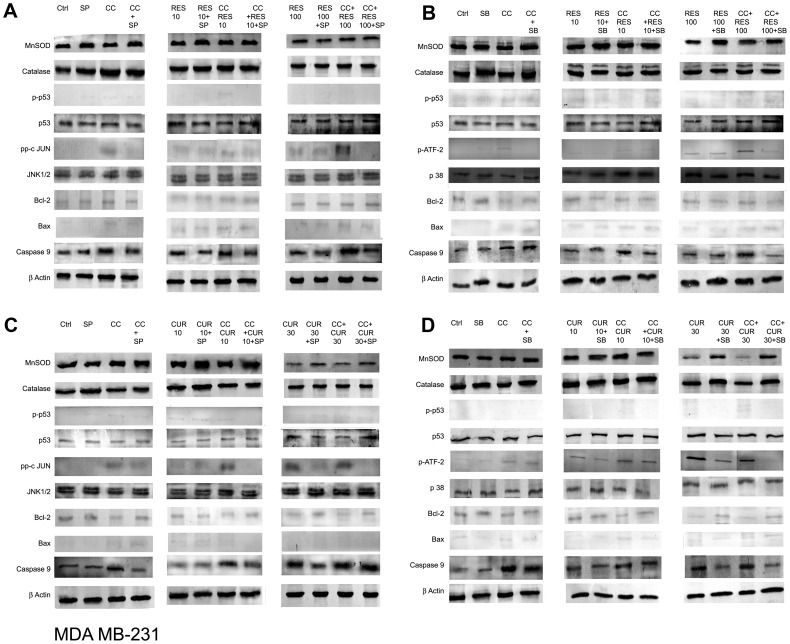
JNK/p38-dependent modulation of expression of Antioxidant enzymes, p53 and apoptogenic factors in MDA MB-231 cells. For gene-product analysis, MDA MB-231 cells were pre-cultured for 48 h in phenol red-free DMEM and exposed to CC (10 µM) and polyphenols *per se* {RES: 10/100 µM (A & B) and CUR: 10/30 µM (C & D)} or sensitized with low/high dose polyphenols for 6 h and continued with CC (10 µM) for next 18 h. Using 10 µM SP600125/1 µM SB 203580, 2 hours prior to CC exposure, 50 µg of the whole cell lysate was separated on a 10% SDS-PAGE gel, probed with respective antibodies following transfer to a nitrocellulose membrane and subsequent immunoblotting. β-actin was used as a protein loading control. (A) RES/CC+RES without and with SP600125; (B) RES/CC+RES without and with SB203580; (C) CUR/CC+CUR without and with SP600125; (D) CUR/CC+CUR without and with SB203580. However, for c-Jun and phospho ATF-2 detection, immunoprecipitation and detection was carried out as detailed in Materials and Methods. Data shown is representative of one of the three similar experiments each performed in triplicate.

A sustained flux of ROS results in an imbalance of the intracellular redox state regulated by Glutathione (GSH), Superoxide Dismutases (SOD) and Catalases (CAT) [22], [23]. RES and CUR both strongly inﬂuenced total GSH dose-dependently where the former down-regulated while the converse was true for the latter in MCF-7 cells (Fig. 4A). However, in MDA MB-231 cells total GSH remained unaltered through dose-dependent RES/CUR (Fig. 4A). The disparity between the relative regulations of GSH in MCF-7 cells may be attributed to structural dissimilarities, extent of conjugation, half-life, stability etc. of the polyphenols in question [24], [25]. This coupled with the other sequel of events may finally determine the fate of the cells. Sensitization with low dose polyphenols in CC treated cells further reduced the total GSH than with ligands alone in MCF-7 cells. Sensitization with 10 µM RES in CC treated MDA MB-231 cells displays unaltered or further reduced total GSH with RES and CUR respectively compared to drugs alone. 100 µM RES sensitized CC treated cells reduced the total GSH compared to CC alone in MCF-7. However, this remains unaltered compared to RES alone in both cell types (Fig. 4A). 30 µM CUR sensitized CC treated cells reduced the total GSH compared to CUR to increase with CC both in MCF-7 and MDA MB231 cells (Fig. 4A). Moreover, compared to control unlike impervious MnSOD expression with low dose polyphenols in both MCF-7/MDA MB-231 cells, the SOD activity enhanced to decline at their respective higher dose. However, in MDA MB-231 cells, polyphenols at both the doses showed equivalent decline (Fig. 4B, 5 and 6). CC alone did not alter the expression although the activity rose in MCF-7 and decreased in MDA MB-231 cells. With 10 µM RES/CUR sensitization, the level and activity has inverse relationship as compared to CC alone in MCF-7 cells. With 10 µM RES/CUR sensitization in CC treated MDA MB-231 cells, MnSOD level remain unaltered while activity declined in both the cases compared to CC *per se*. With high dose polyphenols sensitization, compared to CC or polyphenols alone, reduction (MCF-7) and no alteration (MDA MB-231) was observed (Fig. 4B, 5 and 6). Interestingly, CAT expression remained unaltered throughout the low dose exposure either separately or together versus untreated control cells. However, activity remain unaltered at low doses with RES alone or in sensitized cells, while CUR sensitization in CC treated cells displayed decline in activity as compared to the CUR alone in MCF-7 cells (Fig. 4B and 5). In MDA MB-231 cells, low dose polyphenols down-regulated the activity of CAT which further remained unaltered in sensitized cells as compared to polyphenols alone and decreased vis-à-vis CC (Fig. 4B and 6). Further, CAT expression remains unaltered with high dose polyphenols alone in MCF-7 and decline in MDA MB-231 cells. With high dose polyphenols sensitization, compared to CC or polyphenols alone, reduction and no alteration in activity was observed (Fig. 4B, 5 and 6).

### JNK and p38 Pathway Dependent Differential Expression of Apoptotic Markers in Polyphenol Sensitized, CC Treated MCF-7/MDA MB-231 Cells

**Figure 7:**
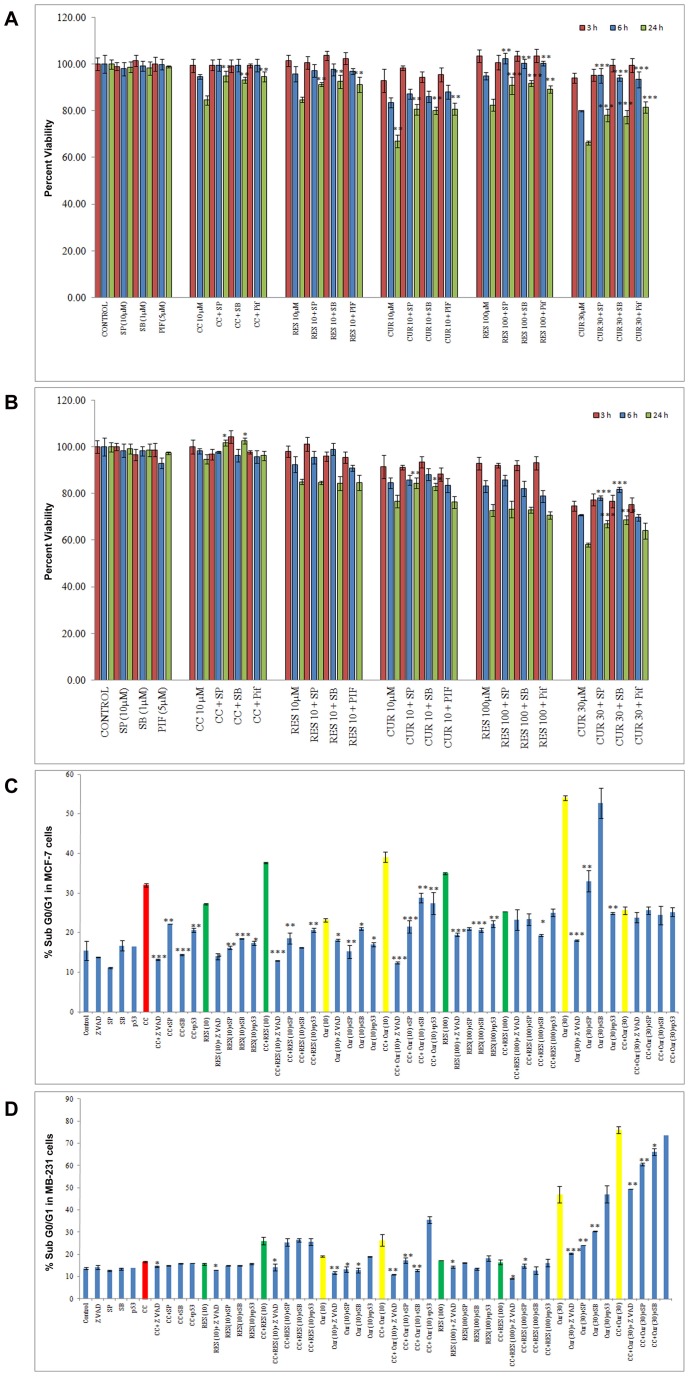
Time-dependent JNK, p38 and p53 pathway inhibition studies induced by CC and polyphenols alone and JNK/p38-dependent apoptosis in sensitized cells. 10^4^ MCF-7 (A)/MDA MB-231 (B) cells were pre-cultured for 48 h in phenol red-free DMEM then exposed to CC (10 µM)/polyphenols *per se* (RES: 10/100 µM and CUR: 10/30 µM) for MTT analysis. Percentage inhibition was determined per the formula {(Absorbance of Control – Absorbance of drug treated cells without and with inhibitor)/Absorbance of Control cells} ×100. For inhibitor experiments through Flow Cytometry, 0.2×10^6^ MCF-7 (C)/MDA MB-231 (D) cells were pre-cultured for 48 h in phenol red-free DMEM, exposed to CC (10 µM)/polyphenols *per se* (RES: 10/100 µM and CUR: 10/30 µM) or preconditioned with low/high dose polyphenols for 6 h and continued with CC (10 µM) for next 18 h. JNK (10 µM SP600125), p38 pathway (1 µM SB 203580), Pifithrin (15 µM Pif) and zVAD-fmk (30 µM) inhibitors were added during the last 2 hours prior to CC exposure, followed by the analysis of the Sub-G0/G1 fraction by FACS. Each data point is the Mean ± S.E. of one of three similar experiments each performed in triplicate. * P<0.05, ** P<0.01, *** P<0.001 drugs compared to drug treatment without inhibitor.

The sequel of events regulating apoptosis vis-à-vis oxidative stress has JNK, p38 and p53 as key SAPKs actors [26], [27]. MTT and FACS studies in polyphenols sensitized CC treated cells employing inhibitors (SP 600125/SB 203580/Pif) were conducted (Fig. 7A–D).

For MTT analysis, 3–24 h exposure with CC/low dose polyphenols slightly inhibited JNK, p38 and p53 pathway by 24 h. However with 100 µM RES and 30 µM CUR, the significant decline of these parameters starts at 6 h until 24 h (Fig. 7A). We therefore propose that at 6 h in MCF-7 cells, the activation of these pathways correlates well with mitochondrial potential supported by elevated ROS. In MDA MB-231 cells, 10 and 100 µM RES displayed no involvement of JNK and p38 pathways, while 10 and 30 µM CUR displayed their involvement at 24 h/6 h until 24 h respectively (Fig. 7B).

Based on the above, for the flow studies on cells during 6 h sensitization with the polyphenols, the inhibitors were individually added during the last 2 h. Subsequently, CC treatment until 18 h was undertaken. Significantly reduced apoptosis through CC and polyphenols exposure *per se* correlating well with MTT studies was observed. More, in case of inhibitors (Z-VAD-FMK, SP 600125/SB 203580/Pif) low dose polyphenols sensitized CC treated cells registered decline in apoptosis to an extent greater than with ligands *per se* in MCF-7 cells. 100 µM RES by itself significantly decreased apoptosis with inhibitors. Notably, with 30 µM CUR plus Z-VAD-FMK/SP 600125/Pif the magnitude of cell death was lower unlike SB 203580 indicating the divergence of pathways at higher molarity. Additionally, the inhibitors fail to exert any effect at high dose of polyphenols sensitized CC treated MCF-7 cells (Fig. 7C). However, p53 null MDA MB-231 cells were expectedly insensitive to p53 inhibitor Pif. In these cells, 10 and 100 µM RES alone as well as in sensitized cells displayed no involvement of JNK and p38 pathways, however caspase-mediated cell death was observed. With 10 and 30 µM CUR alone and in sensitized cells, involvement of Caspases, JNK and p38 pathways was noticed. In case of CC with slight inhibition of apoptosis, other three pathways were unaffected (Fig. 7D).

**Figure 8:**
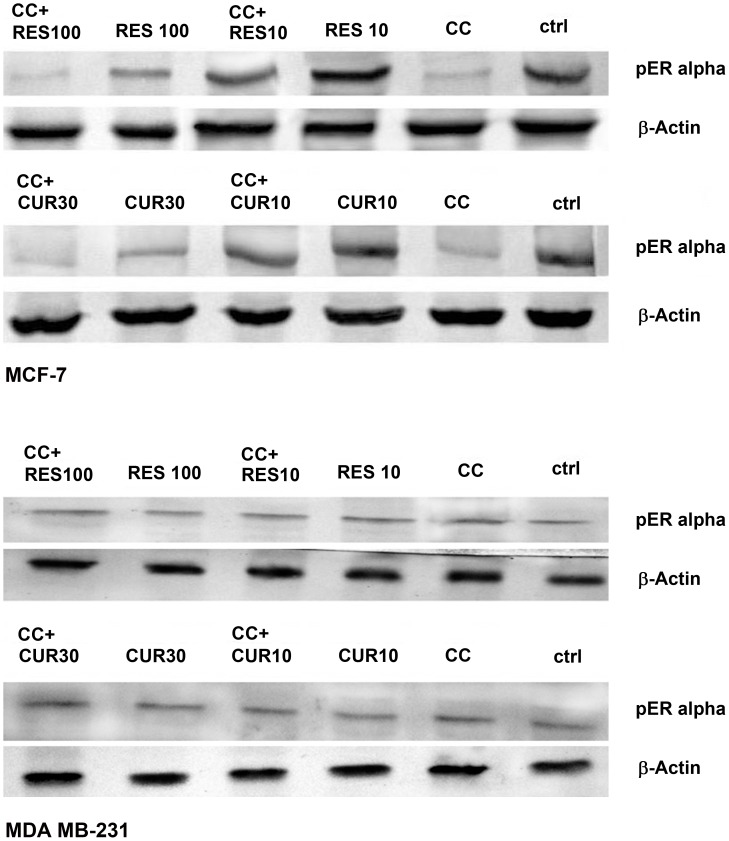
Ser-167 phosphorylation of AF-1 region of ER α. 3×10^6^ MCF-7/MDA MB-231 cells were exposed to CC (10 µM)/polyphenols *per se* (RES: 10/100 µM and CUR: 10/30 µM) or sensitized with low/high dose polyphenols for 6 h and continued with CC (10 µM) for next 18 h. 50 µg of the whole cell lysate was separated on a 10% SDS-PAGE, probed with respective antisera following transfer to a nitrocellulose membrane with immunoblotting using β-actin as control. Data shown is one of three similar experiments each performed in triplicate.

In MCF-7 cells, stress activated protein kinases (SAPKs) expression indicates pan-unaltered total JNK1/2 and p38. Concomitantly, phosphorylation of c-Jun and ATF-2 was up-regulated at low dose polyphenol sensitized CC treated cells versus CC and polyphenols alone. This is supported by the data through respective inhibitors (Fig. 5). With 100 µM RES sensitization phospho c-jun enhanced as compared to drugs alone, while with 30 µM CUR sensitization the expression remains unaltered. The regulation of ROS components is supported by the expression studies of MnSOD and Catalase in the presence and absence of SP 600125 and SB 203580 respectively. The inhibitors up-regulated the expression of MnSOD and Catalase as compared to their respective treatments (Fig. 5). SAPKs down-regulate Ser-167 phosphorylation of ER, a site that influences AF-1 ER-dependent transcriptional activity (non-genomic events) [8], [28]. With low dose polyphenol sensitization, the Ser-167 phosphorylation decreased as compared to polyphenols while reverse was true vis-à-vis CC exposure. However, on sensitization with high dose polyphenols the combination displayed decline in Ser-167 phosphorylation vis-à-vis both the drugs (Fig. 8). The knowledge of stress induced Ser-15 phosphorylation of p53-mediated apoptosis through JNK and p38 SAPKs [2], [15] made us examine the role of SP 600125 and SB 203580 respectively. Compared to untreated control, phosphorylation of p53 was up-regulated dose-dependently with polyphenols and CC alone suppressible by the inhibitors. At low and high dose polyphenols sensitization in CC treated cells, phosphorylation rises and falls respectively compared to either drug. The phosphorylation of p53 in low dose polyphenols sensitized CC treated cells was inhibited both by SP 600125 and SB 203580 which at higher dose remained unaltered or decreased respectively. Total p53 showed constitutive expression (Fig. 5). Phosphorylation of p53 promotes apoptosis whose downstream effectors e.g. Bax/Bcl-2 ratio and caspase-9 [29] examined through western analysis. CC alone and polyphenols dose-dependently up-regulate their activation compared to untreated control. With low dose polyphenols sensitization in CC treated cells, the markers increased to subsequently decrease at higher dose than either drug alone (Fig. 5).

In p53 mutant MDA MB-231 cells [2] the gene regulates Bax expression where total p53, Ser-15 phosphorylation of p53 and Bax was found to be unaltered in CC/RES/CUR exposed cells with or without inhibitors (Fig. 6, A–D). RES dose-dependently did not display any significant increase in the apoptosis (unaltered Bcl-2 and Caspase-9) accompanied with no phosphorylation of c-jun/ATF-2 (Fig. 6, A and B). On sensitization with 10 µM RES in CC treated cells, these factors further remained unaffected with no involvement of JNK/p38 pathway. Conversely, involvement of JNK/p38 pathway through phosphorylation of c-Jun and ATF-2 was evidenced in100 µM RES sensitized CC treated cells versus CC and polyphenols alone (Fig. 6, A and B). However, progressive phosphorylation of c-jun/ATF-2, down-regulation of Bcl-2 and unaltered Caspase-9 was observed dose-dependently in CUR treated cells. Further, on sensitization with low dose CUR in CC treated cells the phosphorylation of c-jun/ATF-2 further rose and was subsequently inhibited both by SP 6000125 and SB 203580 respectively. With 30 µM CUR sensitization, phosphorylation of c-jun/ATF-2 and Caspase-9 were down-regulated compared to CUR alone or remained unaltered vis-à-vis CC respectively. Total JNK/p38 showed constitutive expression. These events were further supported by the data through respective inhibitors. Further, MnSOD and Catalase were also evaluated in presence of SP 6000125 and SB 203580 whose expression remained unaltered compared to respective treatments (Fig. 6, C and D). Finally, the SAPKs regulated Ser-167 phosphorylation of ER alpha in MDA MB-231 cells also displayed no change in expression in CC/RES/CUR alone or in combination treated cells (Fig. 8).

## Discussion

Despite decades of research, the use of dietary antioxidant supplementation during conventional chemo- and radiation therapy remains controversial vis-à-vis the efficacy and safety of complementary treatment [30], [31]. Several *in vitro* studies have demonstrated that the concurrent administration of natural products viz. RES [14], [15], [22], [31], CUR [22], [31]–[32] with chemo- or radiation-therapy reduces treatment-related side effects without/with improving the efficacy of chemotherapy. Here, we determine the role of Centchroman (CC)/Polyphenols alone plus polyphenols sensitized CC treated cells during stress signaling and intracellular redox status *in vitro* using the established Estrogen receptor (ER) positive (+ve) MCF-7 and negative (-ve) MDA MB-231 Human Breast Cancer Cells (HBCCs). Polyphenols beyond pharmacological concentrations (≥10 µM) elicit ER-independent non-genomic effects [33]. Conversely, sub-pharmacological concentration (≤10 µM) induces sub-lethal oxidative stress through ER-dependent non-genomic phenomena [20]. Hence, sensitizing HBCCs with such doses may synergize/antagonize the action of CC. On an overall basis, this may be through the increased generation of Reactive Oxygen Species (ROS) affecting MAPK and similar stress-signaling cascades [33]–[35].

The initial observation that CC significantly causes time-dependent apoptosis (3–24 h) through oxidative stress accomplishing its cytotoxicity in MCF-7/MDA MB-231 cells provided the basis of this study (Fig. 1A and B, 2C, 3A and B). At 24 h, ROS generation (DCFDA) and decreasing ΔΨm triggers subsequent events (Fig. 3A and B). Further, altered thiol, SOD/Catalase ratio modulates redox status, induces SAPKs (JNK/p38) mediating p53-dependent/independent apoptosis in MCF-7 (altered Bax/Bcl-2 ratio, up-regulated caspase-9)/MDA MB-231(absence of Bax, unaltered Bcl-2 and activated Caspase-9) (Fig. 4, 5, 6 and 7A–D). Our previous [2], [3] and current study demonstrates CC mediated up-regulation of JNK and p38 pathways (SAPK) with greater intensity in MCF-7 than MDA MB-231. Therefore, the ratio of MAPK/SAPK is the critical determinant of down-regulation of Ser-167 phosphorylation, a site that influences AF-1 ER-dependent non-genomic transcriptional events in MCF-7 (Fig. 8). However, the MDA MB-231 cells are independent of these events. We therefore, infer that the fold increase of apoptosis was higher in MCF-7 than MDA-MB-231 cells reinforcing the role of receptor and p53 expression [2], [3].

The number of cells in sub-G0/G1 population on treatment with low dose RES/CUR increased significantly by 6 h extending up to 24 h in MCF-7/MDA MB-231 cells (Fig. 2A, B, D and E). However, with RES treated MCF-7 cells, the majority was arrested in S phase (24 h) [36] (Fig. 2A) while in CUR, the cells continued cycling accompanied with significant apoptosis [37] (Fig. 2D). For MDA MB-231 cells, no arrest was observed in both the ligands (Fig. 2B and E). Unlike MDA MB-231 cells, similar phosphorylation of Ser-167 of AF-1 region of ER alpha containing MCF-7 cells through low dose of RES/CUR supports comparable ER-dependent non-genomic transcriptional events compared to control [20] (Fig. 8). Polyphenols induce ROS via SAPK (JNK and p38 MAPK) capable of phosphorylating p53 under cell-cycle arrest or apoptosis is well known for MCF-7 cells [21]. p53 deficient MDA MB-231 cells proceed differently which may explain the dichotomy of apoptosis in the two cell types [2]. In relation to this we found, low dose polyphenol at 3 and 6 h neither showed significant ROS generation nor involvement of JNK, p38, p53 pathways and correlates well with unaltered morphology in MCF-7/MDA MB-231 cells (Fig. 3A and B, 5 and 6, 7A–D, 1A and B). However, at 24 h significant increase of ROS was observed both in MCF-7/MDA-MB-231 cells. RES decreased GSH in MCF-7 or remained unaltered in MDA MB-231 cells. Conversely, CUR enhanced total GSH as compared to control in both cell types as reported previously [25] (Fig. 4A). These events were accompanied with increased MnSOD activity with unchanged CAT in MCF-7 and decreased MnSOD, CAT in MDA MB-231 leads to generation of ROS whose levels do not affect the JNK/p38 signaling as well as mitochondria mediated apoptotic events (Fig. 4, 5 and 6). We therefore, infer that polyphenols pre-incubation sensitizes MCF-7 cells for improved CC action.

The enormity of apoptosis increased significantly in low dose RES/CUR sensitized CC treated MCF-7/MDA MB-231 cells with treatment time. Enhanced S phase arrest (RES sensitized)/apoptosis (CUR sensitized) increases phosphorylation of p53 in MCF-7 cells (Fig. 2 F and G). Simultaneously, increased JNK/p38 and decline of ΔΨm activates downstream events such as Bax/Bcl-2 ratio and caspase-9 (Fig. 3D, 4 and 5). ROS burst with concomitant increase of SOD activity/unaltered CAT indicates altered redox homeostasis in low dose polyphenol sensitized MCF-7 cells (Fig. 3C, 4B). Increased apoptosis (RES sensitized)/G2/M phase arrest (CUR sensitized) in the absence of phosphorylation of p53 in MDA MB-231 cells shows the differential mode of action of the two polyphenols. Unlike RES, CUR showed JNK/p38 mediated apoptosis in MDA MB-231 cells (Fig. 6). The foregoing favorably contributes to the enhanced pro-apoptotic action in CUR sensitized CC treated MDA MB-231 cells as compared to either drug alone. We therefore, deduce that RES/CUR enhances the CC action through ROS mediated JNK/p38 as well as mitochondrial pathway in MCF-7 cells. However, RES/CUR sensitization enhanced apoptosis in MDA MB-231 cells without and with involvement of ROS mediated JNK/p38. Thus, despite being similar, RES and CUR have subtle differences in their molecular mode of action. The review by Howells [31] and references therein have shown evidences for synergistic actions of RES and CUR with various drugs in different cell lines.

RES at 100 µM caused an early (3 h)/late increase (24 h) in ROS (Fig. 3A) reaching its nadir in MCF-7/MDA MB-231 respectively, due to accumulation of cells in G0/G1 phase (Fig. 2A and B) [36], [37]. Contrarily, with 30 µM CUR, the enhanced ROS at 3 h was retained up to 24 h owing to cell arrest in G2/M phase in both cell types (Fig. 3A) [38], [39]. The direct relationship between intracellular-redox cybernetics/G0/G1 and G2/M arrest and activation of MAP kinases-mediated phosphorylative pathways has been demonstrated [40]–[42]. Here we report that, 100 µM RES/30 µM CUR preferentially causes significant disruption of ΔΨm (Fig. 3B), inhibition of AO enzymes expression and induction of JNK/c-Jun and p38/p-ATF2-mediated phosphorylation in MCF-7 cells (Fig. 5) [21], [35]–[37], [39], [41]. Unlike 100 µM RES, 30 µM CUR showed JNK/p38 mediated apoptosis in MDA MB-231 cells (Fig. 6, 7B and D) [42], [43]. Therefore, both reduced antioxidant capacity and selective activation of different MAP kinases act as the principal mediators of apoptosis (altered ratio of Bax/Bcl-2 ratio and Caspase-9 activation) in MCF-7 cells (Fig. 6) [21], [36], [44], [45] and MDA MB-231 (decreased Bcl-2 and activation of Caspase-9, Fig. 6) [42], [43]. At the molecular level, we conclude and others support that ER-independent non-genomic events (down-regulation of Ser-167 phosphorylation of AF-1 region of ER) are prominent with higher dose of polyphenols in MCF-7 [7] but not in MDA MB-231 cells (Fig. 8). The data discussed above has been correlated with concomitant time-dependent studies employing JNK/p38/p53 inhibitors (Fig. 7A and B). The above events along with altered redox status sensitize the cells for CC mediated apoptosis following 3–6 h polyphenols treatment.

Nonetheless, sensitization with high dose polyphenols renders the cells under arrest in Go/G1 (RES)/G2/M phase (CUR) as early as 3 h in MCF-7 versus 24 h in MDA MB-231cells preventing CC action and resultant apoptosis (Fig. 2F and G). This thereby leaves the factors unaltered in CC treated polyphenols sensitized cells than with polyphenols *per se* in both cell types. Interestingly, CC plus RES displayed the involvement of JNK/p38 pathways in MDA MB-231 cells (Fig. 6). This also implies that high dose polyphenols by themselves invoke cell death. This may be however, unachievable physiologically owing to poor bioavailability etc. hence is more of academic value. Efforts therefore should be directed towards modifying the structure of polyphenols to overcome this paradox.

Therefore, low dose polyphenols sensitization with demonstrated safety vis-à-vis normal cells can augment apoptotic efficacy of hormonal therapeutics like CC in ER-positive/negative MCF-7/MDA MB-231 cells. Hence, sensitization through polyphenol under *in vivo* situation may improve the efficacy of hormonal-therapy with reduced treatment-related morbidity.
